# *MAGE* A1-A6 RT-PCR and *MAGE* A3 and *p16* methylation analysis in induced sputum from patients with lung cancer and non-malignant lung diseases

**DOI:** 10.3892/or.2011.1566

**Published:** 2011-11-29

**Authors:** KYEONG-CHEOL SHIN, KWAN-HO LEE, CHAE-HUN LEE, IM-HEE SHIN, HUN-SUK SUH, CHANG-HO JEON

**Affiliations:** 1 Department of Internal Medicine, College of Medicine, Yeungnam University, Daegu; 2 Department of Laboratory Medicine, College of Medicine, Yeungnam University, Daegu; 3 Department of Medical Statistics, College of Medicine, Catholic University of Daegu, Daegu, Republic of Korea; 4 Department of Laboratory Medicine, College of Medicine, Catholic University of Daegu, Daegu, Republic of Korea

**Keywords:** lung cancer, sputum, melanoma antigen gene RT-PCR, melanoma antigen gene A3, *p16*, methylation

## Abstract

The melanoma antigen gene (*MAGE*) A1-A6 RT-PCR system was developed for the detection of lung cancer cells in the sputum. However, we identified *MAGE* expression in some patients with non-malignant lung diseases. To understand these patterns of *MAGE* expression, we performed *MAGE* A3 methylation-specific PCR (MSP) and *p16* MSP. We collected 24 biopsy specimens of lung cancer tissue and performed *MAGE* A1-A6 RT-PCR, *MAGE* A3 MSP and *p16* MSP. RNA and DNA were simultaneously extracted from induced sputum specimens of 133 patients with lung diseases and 30 random sputum specimens of healthy individuals and the 3 molecular analyses were performed. The patients were diagnosed as 65 cases of lung cancer and 68 of benign lung diseases. Positive rates of *MAGE* A1-A6 RT-PCR, *MAGE* A3 MSP and *p16* MSP were as follows: in lung cancer tissue, 87.5, 58.3 and 70.8%; in the sputum of lung cancer patients, 50.8, 46.2 and 63.1%; benign lung diseases, 10.3, 30.9 and 39.7%; and healthy individuals, 3.3, 6.7 and 3.3%. Of the 40 *MAGE*-positive cases, 33 were diagnosed with lung cancer and 7 as having benign lung diseases. From the 7 cases of *MAGE*-positive benign lung diseases, 6 cases showed methylation abnormalities. The *MAGE*-positive group revealed significantly higher rates of methylation abnormalities. Of the 40 *MAGE*-positive cases, 39 cases were found to be lung cancer or benign lung diseases with abnormal methylation. Thus, *MAGE* expression in the sputum suggests the presence of lung cancer cells or pre-cancerous cells.

## Introduction

For the detection of lung cancer cells in the sputum, we developed melanoma associated gene (*MAGE*) primers that can amplify *MAGE* A1-A6 simultaneously (1), and evaluated the positive rates in induced sputum of patients with lung cancer (2). Meanwhile, we detected *MAGE* expression not only in patients with lung cancer but also in some patients with non-malignant lung diseases, including tuberculosis and inflammation. Expression of *MAGE* by de-methylation of the *MAGE* promoter region has been demonstrated (3), and its expression was known to be restricted to germ cells and cancer cells (4,5). However, Mecklenburg *et al* reported that *MAGE* expression was also detected in patients with inflammatory diseases (6).

As inflammation is a critical component of tumor progression, repeated and chronic inflammation can increase cancer risk (7). Considering the course of lung carcinogenesis, before which molecular biological change has occurred, *MAGE* expression in the sputum of inflammatory disease can be regarded as a reasonable event. However, applying this result to clinical diagnosis can increase the probability of false positive diagnoses and lead to difficulties in patient care. For the clinical application of *MAGE* reverse transcription polymerase chain reaction (RT-PCR), it would be necessary that the *MAGE* result be carefully interpreted along with additional molecular findings and clinical evidence.

This study was designed to evaluate the performance of *MAGE* RT-PCR, *MAGE* A3 methylation-specific PCR (MSP), and *p16* MSP using induced sputum of patients with lung cancer or non-cancerous inflammatory diseases, and to compare the results of genetic tests with the patients' clinical findings. Eventually, we attempted to elucidate the clinical significance of *MAGE* expression in the sputum of patients with pulmonary diseases.

## Subjects and methods

### Subjects

Twenty-four biopsy specimens were obtained from patients with lung cancer who underwent bronchoscopy at Yeungnam University Hospital from 2006 to 2008. Obtained tissues were immersed immediately in TRI solution (Molecular Research Center, Cincinnati, OH), and stored in a deep freezer until RNA extraction.

During the same period, 133 samples of induced sputum specimens collected from patients with pulmonary problems who visited Yeungnam University Hospital were added to sputum RNA extraction solution (iC&G Co., Daegu, Korea), and stored in a -70°C refrigerator until required for RNA extraction. Induced sputum was obtained after inhaling Berotec solution (Boehringer Ingelheim, Ingelheim, Germany) and 16 ml of 3% hypertonic saline. To determine the clinical diagnosis for the patients, history taking, physical examinations, bronchoscopy, computed tomography (CT) scan, and histopathological biopsies were performed.

Random sputa were collected from 30 healthy volunteers, and treated equally. All stored specimens were blindly transferred to the Department of Laboratory Medicine at Daegu Catholic University Medical Center, then *MAGE* A1-A6 RT-PCR, *MAGE* A3 MSP, and *p16* MSP were carried out. Tissue and sputum procurement procedures were approved by the Institutional Review Board of the Yeungnam University Hospital. Informed consent was obtained from all patients.

### RNA and DNA extraction and RT-PCR

RNA and DNA extraction from sputum specimens was conducted with an iC&G extraction kit using magnetic beads. RNA from tissues was extracted according to the TRI Corporation's instructions, and its DNA was extracted using TRI remnant. RNA was reverse-transcribed using ImProm-II reverse transcription (RT) reagents (Promega Corp., Madison, WI), and *MAGE* A1-A6 expression was amplified using iC&G PCR reagents. cDNA integrity was confirmed by *GAPD* amplifications.

### Methylation-specific PCR

**Figure 1:**
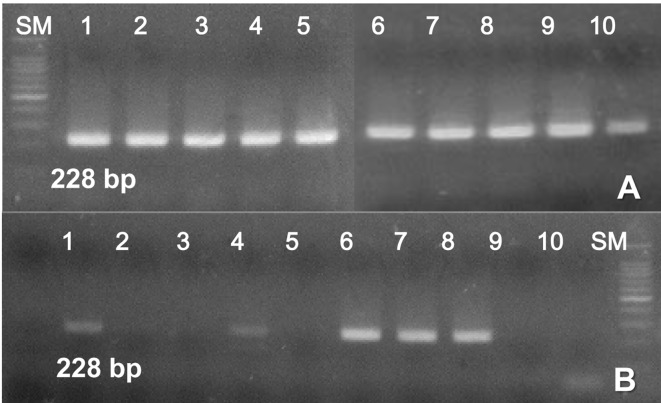
*MAGE* A3 methylation specific PCR (MSP) results in sputum of patients with lung diseases. (A), *MAGE* A3 MSP; (B), *MAGE* A3 unmethylation specific PCR; SM, Size marker; lanes 1-10, number of patient's matched specimen.

**Figure 2:**
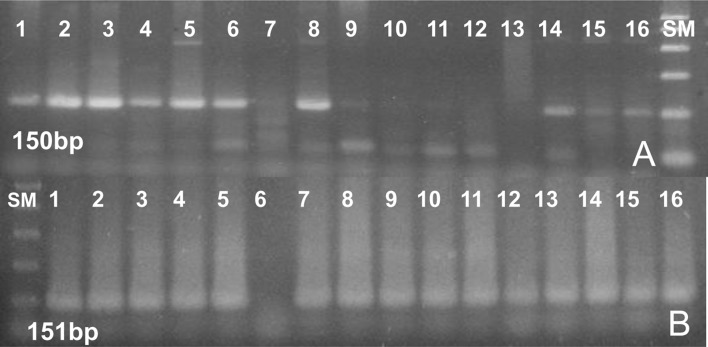
p16 methylation specific PCR (MSP) results in the sputum of patients with lung diseases. (A), *p16* MSP; (B), *p16* unmethylation specific PCR; SM, size marker; lanes 1-16, number of patient's matched specimen.

Unmethylated cytosine was changed to uracil in the CG nucleotide gene of extracted DNA using Cp genome change reagent (Chemicon, Temecula, CA). Nested PCR was performed on treated genomic DNA for amplification of *MAGE* A3 and *p16* MSP. At first, the target gene was amplified for 30 cycles, regardless of methylation. Then, MSP was performed for 30 cycles using methylation-specific primers and Gold Taq enzyme (Perkin-Elmer, Boston, MA). Cp™ genome universal methylated DNA (Chemicon) was used as a positive control sample of *MAGE* A3 and *p16* MSP. Primer sets for *MAGE* A3, *p16* MSP, and *GAPD*, annealing temperatures and product sizes are shown in Table I. The amplified products of *MAGE* A3 MSP, and *p16* MSP were clearly visualized in Fig. 1 and Fig. 2, respectively.

### Statistical analysis

The repeated-measure one factor analysis of the Cochran test was performed to compare the positive rates of *MAGE* A1-A6 RT-PCR, *MAGE* A3 MSP, and *p16* MSP. The Chi-square test was used for comparison of positive rates among the patients' groups. Statistical analyses were conducted using SPSS 14.0 software (SPSS Inc., Chicago, IL). A P-value of <0.05 was considered to indicate statistical significance.

## Results

### Subjects

The 133 enrolled patients were diagnosed as 65 lung cancer and 68 benign lung diseases. Patients of benign lung diseases were classified as follows: 25 no active lung disease, 16 pulmonary tuberculosis, 11 pneumonia, 11 inflammatory diseases, 2 bullae, 2 pleural effusion of unknown cause, and 1 right middle lobe syndrome.

The mean ages of patients with lung cancer, patients with benign lung diseases, and healthy volunteers were 66.0±12.7, 59.0±15.6 and 29.5±9.79 years, respectively. The gender distributions of these groups were 8.29:1, 5.18:1, and 0.58:1, respectively.

### Positive rates for MAGE A1-A6 RT-PCR, MAGE A3 MSP, and p16 MSP according to the patient group

Positive rates for *MAGE* A1-A6 RT-PCR, *MAGE* A3 MSP, and *p16* MSP were as follows. In tissues of patients with lung cancer, 87.5, 58.3, and 70.8%; in induced sputa of patients with lung cancer, 50.8, 46.2, and 63.1%; in induced sputa of patients with benign lung disease, 10.3, 30.9, and 39.7%; in random sputa of healthy people, 3.3, 6.7, and 3.3%. All 3 tests showed statistically significant results using the sputum of lung cancer, benign diseases, and healthy groups (P<0.05) (Table II). In the group of lung cancer, *MAGE* RT-PCR revealed statistically significant higher positive rates while in benign lung diseases, significant lower positive rates than *MAGE* A3 and *p16* MSP results (P<0.05).

### Positive rates for MAGE RT-PCR, MAGE A3 MSP, and p16 MSP in patients with benign lung diseases

*MAGE* expression rates were high in patients with inflammatory diseases and tuberculosis (18.2 and 18.8%, respectively), while positive rates for *MAGE* A3 unmethylation and *p16* methylation were high in patients with tuberculosis (56.3 and 62.5%) and in those with pneumonia (45.5 and 54.5%). The average positive rate of *MAGE*, *MAGE* A3 unmethylation, and *p16* methylation in patients with benign lung diseases were 10.3, 30.9, and 39.7% (Table III), showing a statistically significant difference (Table II).

### Positive rates for MAGE A3 MSP and p16 MSP in regard to MAGE expression

**Figure 3:**
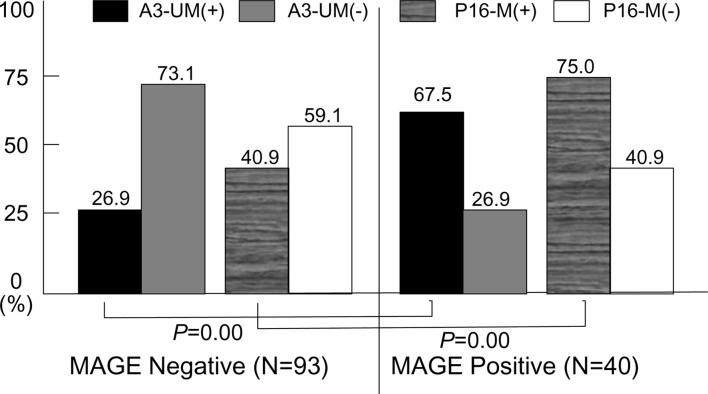
Positive rates of *MAGE* A3 and *p16* methylation-specific PCR according to *MAGE* results in the sputum. The Chi-square test was performed to compare the positive rates.

In the 40 *MAGE* positive cases of lung cancer and benign lung diseases, positive rates for *MAGE* A3 unmethylation and *p16* methylation were 67.5 and 75.0%, respectively. These results showed significant differences compared with the 26.9% positive rates of *MAGE* A3 unmethylation and 40.9% of *p16* methylation in the 93 *MAGE*-negative cases (Fig. 3).

### Clinical analysis and methylation abnormality in the MAGE-positive patients

Of the 40 *MAGE*-positive cases, 33 were diagnosed as lung cancer and 7 as benign lung diseases. The diagnosis of 7 benign lung diseases was as follows: 3 pulmonary tuberculosis, 2 inflammatory diseases, 1 bullae, and 1 no active lung disease. From the 7 cases of benign lung diseases, 5 showed methylation abnormality in both *MAGE* A3 and *p16*. One inflammation showed methylation abnormality in *p16* only and 1 bullae showed no methylation abnormality in either gene (Table IV).

## Discussion

Mortality of lung cancer is still high; therefore, early detection of lung cancer is a major issue. The advent of low-dose spiral chest CT, positron emission tomography, and autofluorescence bronchoscopy (AFB) have opened a new perspective for early detection (8), and specific molecular markers have also been developed (9). Although AFB can detect pre-invasive lesions and lung cancers in the central airway, the specificity of AFB is low (10). A diagnostic test with high sensitivity and high specificity has not yet been developed.

*MAGE* is a highly specific tumor marker, and *MAGE* A3 expression was observed in 35% of lung cancer patients through a multi-center study (11). Based on these findings, *MAGE*-A3 vaccination has been developed as a promising treatment modality for lung cancer (12,13). Atanackovic *et al* (12) reported that 14 of 18 lung cancer patients with stage I or stage II disease had no evidence of disease up to 3 years after vaccination with *MAGE* A3-protein. In addition to *MAGE* A3, a DNA methylation-based biomarker is considered a rapid and efficient early detection marker for lung cancer (9,14), and a previous study reported that DNA methylation marker targeting 4 genes in sputum showed 94% sensitivity and 90% specificity (14). Anglim *et al* demonstrated that aberrant promoter methylation of *p16* was observed 3 years before diagnosis of squamous cell carcinoma in smokers (9).

In the present study, *MAGE* gene-positive rates were 87.5% in lung cancer tissues, 50.8% in induced sputum specimens of patients with lung cancer, 10.3% in induced sputum specimens of patients with benign lung diseases, and 3.3% in random sputum specimens of healthy people. The finding of *MAGE* expression in benign lung diseases is consistent with those of an earlier study, which reported that *MAGE* A1 or A2 expression is detected in severe bronchitis and severe actinomycosis with concomitant tissue regeneration (6). *MAGE* expression was considered an early event in lung carcinogenesis (3) and was detected in precancerous lesions. Therefore, *MAGE* can be used not only as an early detection marker for lung cancer but also as a prevention marker for lung carcinogenesis. In this study, *MAGE* positive benign lung diseases were mainly comprised of pulmonary tuberculosis and inflammatory diseases and severity of inflammation was not evaluated. Kim *et al* (15) reported *MAGE* expression in tissue samples obtained using a percutaneous needle aspiration biopsy of tuberculosis patients. We performed *MAGE* A1-A6 nested PCR using DNA of *Mycobacterium tuberculosis*; however, the *MAGE* gene was not amplified. Therefore, *MAGE* expression of tuberculosis patients may be not the result of a false-positive detection by *Mycobacterium tuberculosis* but the result of the inflammatory process. Compared with the non-tuberculosis group, tuberculosis patients showed coexistence of lung cancer and high incidence rates of lung cancer (16,17).

However, determination of *MAGE* expression in the sputum based simply on clinical conditions cannot reflect molecular biological change at the cellular level. To reflect molecular biological change, additional molecular biological tests associated with lung carcinogenesis are necessary. In order to conduct a proper mutation analysis, a number of tumor cells and a large number of genetic loci should be investigated. Aberrant methylation of the *p16* promoter is an important mechanism of lung carcinogenesis (14) and unmethylation of the *MAGE* A3 promoter is directly associated with *MAGE* expression. Therefore, we performed *MAGE* A3 and *p16* MSP. The MSP is a promising method for detection of lung cancer because it can detect a small number of cancer cells in sputum that contains a large number of normal cells (9).

In the present study, unmethylation rates for *MAGE* A3 MSP were 46.2% in sputa of patients with lung cancers, 30.9% in patients with benign lung diseases, and 6.7% in healthy people. Though unmethylation rates for *MAGE* A3 MSP have not yet been reported in the literature review, these findings are similar to the results of Olaussen *et al* (18), which reported that positive rates for *MAGE* A1 MSP were 50% in cytologically negative sputum from lung cancers patients, 45% in sputum showing inflammatory change from smokers, and 6% in cytologically negative sputum from smokers.

Positive rates for *p16* MSP were 63.1% in sputa of lung cancer patients, 39.7% in patients with benign lung diseases, and 3.3% in healthy people. Olaussen *et al* (18) also reported that positive rates for *p16* MSP were 27% in cytologically negative sputum from lung cancers patients, 64% in sputum showing cancerous cytology from smokers, 27% in sputum showing inflammatory change from smokers, and 47% in sputum showing normal cytology from smokers. Among studies performed in the Korean population, one study reported that *p16* methylation was detected in 67% of tumor samples (19). However, another study reported 22% positive rates in tumor samples, and 1% in the corresponding non-malignant lung tissues (20). The methylation rate of *p16* in lung cancer tissues was about 80% (21,22). Liu *et al* (22) reported methylation rates of 74.7% in the sputum from lung cancer patients, and 51.4% from people exposed to coal smoke. Using matched specimens from lung cancer patients, Hsu *et al* (23) reported 37% methylation rates in lung cancer tissues, 33% in sputa, 13% in normal lung tissues, and 14% in sputa from the control group. Although reported rates for *p16* methylation were variable, *p16* MSP was regarded as a useful molecular marker for use in early detection and prediction of lung cancer.

Therefore, analysis of molecular abnormality in the same specimen using *MAGE* A1-A6 RT-PCR, *MAGE* A3 MSP, and *p16* MSP simultaneously may be very useful for detection and prediction of lung cancer at the molecular level. This analysis can also be applied to non-cancerous groups to understand the clinical significance of *MAGE* expression. In this and other studies, positive rates of MSP remain high in the non-cancerous group; therefore, abnormality of MSP was just utilized as a supplemental modality to explain *MAGE* expression in the sputum, not as a cancer detection tool.

The *MAGE*-positive group in sputum showed a statistically significant higher *MAGE* A3 unmethylation and *p16* MSP methylation rate than the *MAGE* negative group. From the 7 cases of benign lung diseases with *MAGE* expression, 5 showed methylation abnormality in both *MAGE* A3 and *p16*, 1 case showed methylation abnormality in *p16* only, and 1 case showed methylation abnormality in neither *MAGE* A3 nor *p16*. Although the *MAGE* A3 gene could not be expressed without unmethylation of *MAGE* A3, another gene could be amplified because common primers that can amplify *MAGE* A1-A6 together had been used. Moreover, *MAGE* A3 MSP is not a quantitative MSP of promoter loci, but reflects methylation status of primer binding sites. Therefore, the results of MSP may not be consistent with the results of RT-PCR.

The question of how to interpret *MAGE* expression in patients with benign lung diseases is an important issue. *MAGE* proteins form complexes with KAP1, suppress p53-dependent apoptosis, and contribute to cancer development (24). We analyzed *MAGE* expression acting as a tumor enhancer, and also detected the methylation status of *MAGE* A3 and *p16* using the same specimens. Of 40 *MAGE* positive cases in the sputa, 39 cases turned out to be lung cancers or benign lung diseases accompanying methylation abnormality. Therefore, *MAGE* expression in the clinical specimen may suggest the presence of cells in the process of molecular carcinogenesis, even if cancer cells are not visible.

Thus, the clinical significance of *MAGE* expression in the sputum would be i) the presence of lung cancer cells or ii) pre-cancerous cells. In another study (25), we evaluated *MAGE* expression in the peritoneal washes of gastric carcinoma patients, and the patients were followed up for 5 years in regard to *MAGE* expression. As a result, recurrence rates of *MAGE*-positive cases (45.5%) were much higher than those of *MAGE* negative cases (9.6%). Although gastric carcinogenesis may not be identical with lung carcinogenesis, *MAGE* expression in clinical specimens can be considered a strong indication of tumor recurrence. However, not all precancerous cells will develop into cancer, and *MAGE* expression was regarded as a reversible change. Thus, *MAGE* expression in specimens of non-malignant patients should be interpreted very carefully, and for proper clinical application, other clinical information, such as 5-year follow-up results would be studied.

In 2001, Jang *et al* (3) predicted that the *MAGE* gene would be utilized as a tool for lung cancer prevention. Since then, functions of the *MAGE* gene as a tumor promoter have been disclosed, and tumor therapeutic agents targeting the *MAGE* gene have been developed and will soon be applied to lung cancer treatment. The recurrence rate of *MAGE*-positive cases in peritoneal washes of gastric carcinoma patients was 45.5%. In the present study, the *MAGE* A3 unmethylation or *p16* methylation abnormality was demonstrated in *MAGE*-positive specimens from non-cancerous patients. Based on these findings, *MAGE* expression in the sputum may indicate the presence of lung cancer cells or pre-cancerous cells. Therefore, a *MAGE* positive case in the non-cancer group should be closely followed up. In conclusion, *MAGE* could be utilized as a cancer prediction tool as well as a cancer detection tool. Further studies including molecular markers, histological examination, and clinical studies targeting non-cancerous patients with *MAGE* expression are inevitable.

## Figures and Tables

**Table I:** Primer sequences used for *GAPD* PCR, *MAGE* A3, and *p16* methylation specific PCR.

Target	Direction	Sequences (5′→3′)	Ta (°C)	Size (bp)
*GAPD*	F	tcg gag tca acg gat ttg gtc gta	59	320
	R	caa atg agc ccc agc ctt ctc ca		
*MAGE* A3	Outer F	tgt tcg gaa ttt agg gta gta tcg	56	417
	Outer R	ttc cct ctc gaa atc cta acc tta		
	IF (UM)	tgt ttt gag taa tga gtg at	56	228
	IR (UM)	act aaa aca aca aaa atc aac a		
	IF (M)	cgt ttt gag taa cga gcg ac	56	228
	IR (M)	act aaa acg acg aaa atc gac g		
*p16*	Outer F	ggt gtt ata ttc gtt aag tgt tcg	56	482
	Outer R	cta cct aat tcc aat tcc cct aca		
	IF (UM)	tta tta gag ggt ggg gtg gat tgt	65	151
	IR (UM)	c aac ccc aaa cca caa cca taa		
	IF (M)	tta tta gag ggt ggg gcg gat cgc	65	150
	IR (M)	gac ccc gaa ccg cga ccg taa		

F, forward; R, reverse; IF, inner forward; IR, inner reverse; UM, unmethylated; M, methylated.

**Table II:** Positive rates of *MAGE* RT-PCR, *MAGE* A3, and *p16* methylation specific PCR according to the diagnosis.

Specimen	Diagnosis	n	*MAGE* (%)	A3-UM (%)	*p16*-M (%)	P-value^a^
Tissue	Lung cancer	24	87.5	58.3	70.8	0.058
Induced sputum	Lung cancer	65	50.8	46.2	63.1	0.040
Induced sputum	Benign diseases	68	10.3	30.9	39.7	0.000
Random sputum	Healthy people	30	3.3	6.7	3.3	0.607
P-value^b^			0.00	0.00	0.00	

UM, unmethylated; M, methylated;

a The repeated-measure one factor analysis of Cochran test was performed to compare the positive rates of 3 tests;

b The Chi-square test was used for comparison of positive rates among the patient groups.

**Table III:** Positive rates of *MAGE* RT-PCR, *MAGE* A3, and *p16* methylation specific PCR in sputum of patients with benign lung diseases.

Diagnosis	n	*MAGE* (%)	A3-UM (%)	*p16*-M (%)
No active lung disease	25	4.0	16.0	24.0
Inflammatory diseases	11	18.2	18.2	36.4
Tuberculosis	16	18.8	56.3	62.5
Pneumonia	11	0.0	45.5	54.5
Others^a^	5	20.0	20.0	20.0
Total	68	10.3	30.9	39.7
P-value (Chi-square test)		0.306	0.047	0.094

UM, unmethylated; M, methylated;

a 2 cases of bullae, 2 cases of unknown pleural effusion, 1 case of RML syndrome.

**Table IV:** Clinical diagnosis and methylation-specific PCR results for 7 *MAGE*-positive cases of benign lung diseases.

Case no.	Age	Gender	Diagnosis	A3-UM	*p16*-M
1	53	M	No active lung disease	Positive	Positive
2	62	M	TB pleurisy	Positive	Positive
3	77	M	Pulmonary TB	Positive	Positive
4	48	M	Pulmonary TB	Positive	Positive
5	52	F	Inflammation	Positive	Positive
6	74	M	Inflammation	Negative	Positive
7	58	M	Bullae	Negative	Negative

UM, unmethylated; M, methylated.
